# Supplementary figures and images for: Machine learning in predicting respiratory failure in patients with COVID-19 pneumonia—Challenges, strengths, and opportunities in a global health emergency
Source: PLoS One. 2020 Nov 12;15(11):e0239172. doi: 10.1371/journal.pone.0239172 (PMC7660476; doi:10.1371/journal.pone.0239172)

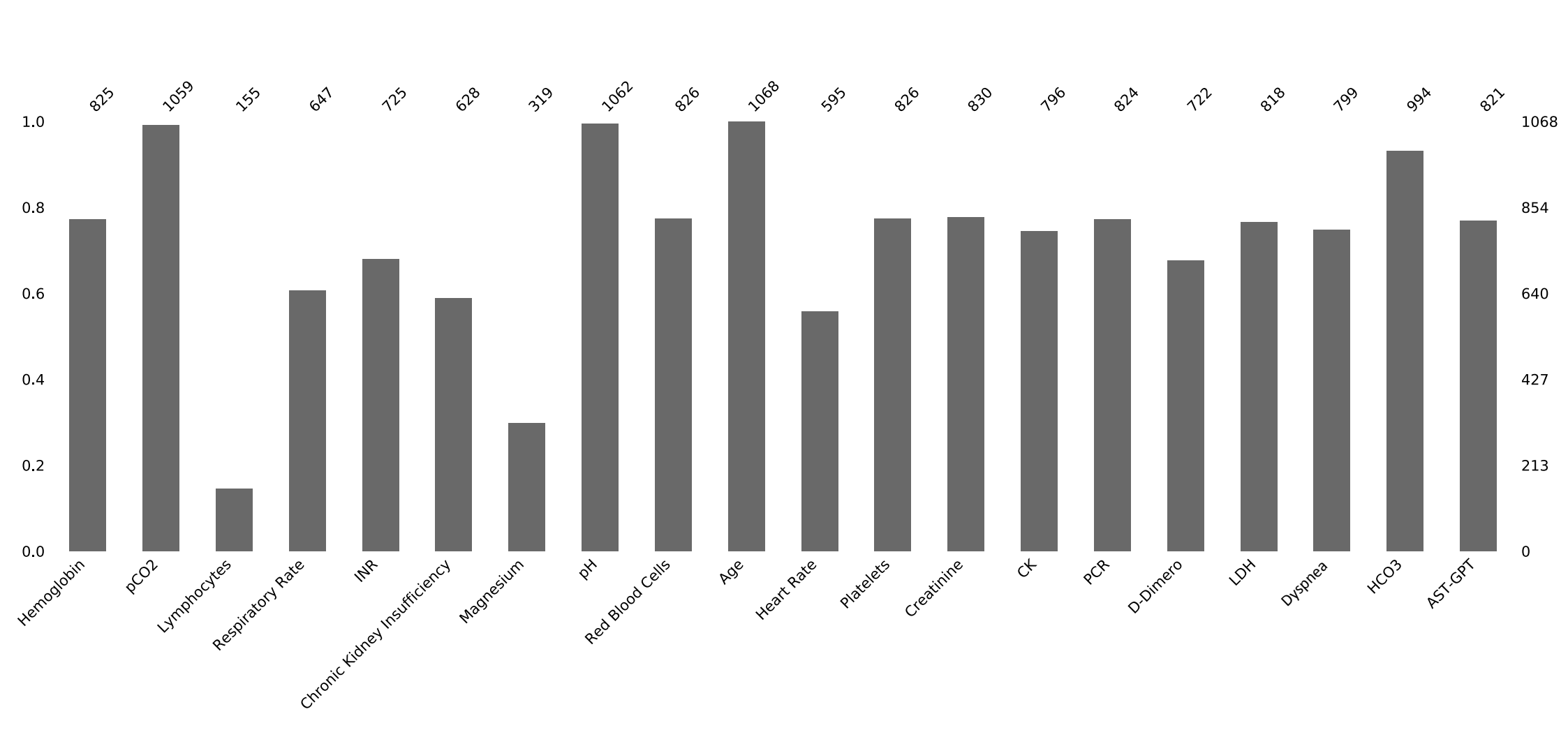

Supplement: S1 Fig — (PDF) [file pone.0239172.s001.pdf]
