# Supplementary material for: Machine learning in predicting respiratory failure in patients with COVID-19 pneumonia—Challenges, strengths, and opportunities in a global health emergency
Source: PLoS One. 2020 Nov 12;15(11):e0239172. doi: 10.1371/journal.pone.0239172 (PMC7660476; doi:10.1371/journal.pone.0239172)
Supplement: S1 File — (DOCX) [file pone.0239172.s002.docx]

# EACH MODEL FEATURES

## Model 1

Age

Conjunctivitis

Cough

Cough with pain

Cough with sleep disturb

Cough with weakness

Cough, breathless then bend over

Cough, breathless when talking

Cough, physical weakness

Diarrhea

Dispnea

Dyspnea while sitting or lying

Dyspnea while walking

Dyspnea while washing or dressing up

Diastolic Pressure

Fatigue

Headache

Heart Rate

Hemoptysis

Lymphadenomegaly

Male

Myalgia

Non Purulent Expectorate

Pharyngitis

Purulent Expectorate

Rash

Respiratory Rate

Rhinorrhoea

Shivers

Systolic Pressure

Tonsillar Edema

## Model 2

Absolute Lymphocytes

ALT-GLT

AST-GPT

Blood glucose

BNP

Calcium

Chlorine

CK

Creatinine

Creatinine in urine

D-Dimero

Ferritin

Fibrinogen

HCO3

Hematocrit

Hemoglobin

INR

Interleukin 6

Lactates

LDH

Lymphocytes

Magnesium

Neutrophils

pCO2

PCR

pH

Platelets

Potassium

Procalcitonin

Procalcitonin PCT

Proteins in urine

PT

Red Blood Cells

Sodium

Total Bilirubin

Transferrin

Troponin

Urea

White Blood Cells

## Model 3

Age

ALT-GLT

AST-GPT

Blood glucose

BNP

BPCO

Calcium

Chlorine

Chronic Kidney Insufficiency

CK

Conjunctivitis

Copatologies

Cough

Cough with pain

Cough with sleep disturb

Cough with weakness

Cough, breathless then bend over

Cough, breathless when talking

Cough, physical weakness

Creatinine

Creatinine in urine

CVD

D-Dimero

Diabetes

Diarrhea

Dispnea

Dyspnea while sitting or lying

Dyspnea while walking

Dyspnea while washing or dressing up

Diastolic Pressure

Hematological Diseases

Emocromo

Fatigue

Ferritin

Fibrinogen

HCO3

Headache

Heart Rate

Hematocrit

Hemoglobin

Hemoptysis

Hepatitis B

Hepatitis C

HIV

Hypertension

INR

Interleukin 6

Iron

Lactates

LDH

Lymphadenomegaly

Linfociti_Assoluti

Liver Insufficiency

Lymphocytes

Magnesium

Magnesium in urine

Male

Myalgia

Neoplasms

Neutrophils

Non Purulent Expectorate

Obesity

Organ Transplant

Other Copatologies

pCO2

PCR

pH

Pharyngitis

Platelets

Potassium

Procalcitonin

Procalcitonin PCT

Proteins in urine

PT

Purulent Expectorate

Rash

Red Blood Cells

Respiratory Rate

Rhinorrhoea

Shivers

Sodium

Sodium in urine

Systolic Pressure

Tonsillar Edema

Total Bilirubin

Transferrin

Troponin

UK

Urea

VES

White Blood Cells

## Model 4

Age

AST-GPT

Chronic Kidney Insufficiency

CK

Creatinine

D-Dimero

Dispnea

HCO3

Heart Rate

Hemoglobin

INR

LDH

Lymphocytes

Magnesium

pCO2

PCR

pH

Platelets

Red Blood Cells

Respiratory Rate
